# Cortical spreading depolarizations in stroke: Mechanisms, neuroprotective interventions, and monitoring techniques

**DOI:** 10.1007/s11357-025-01988-w

**Published:** 2025-11-26

**Authors:** Andrea Díaz-Pérez, Nerea Alvarez de Eulate, Eduard Masvidal-Codina, Xavi Illa, Xavier Navarro, Anton Guimerà-Brunet, Francesc Jiménez-Altayó, Clara Penas

**Affiliations:** 1 Department of Pharmacology, Therapeutics, and Toxicology, Universitat Autònoma de Barcelona, Cerdanyola del Vallès, Spain; 2 Department of Cell Biology, Physiology and Immunology, Universitat Autònoma de Barcelona, Cerdanyola del Vallès, Spain; 3 Institute of Neurosciences, Universitat Autònoma de Barcelona, Cerdanyola del Vallès, Spain; 4 Instituto de Microelectrónica de Barcelona, IMB-CNM (CSIC), Campus UAB, Bellaterra, Spain; 5 Centro de Investigación Biomédica en Red en Bioingeniería, Biomateriales y Nanomedicina (CIBER-BBN), Madrid, Spain; 6 Catalan Institute of Nanoscience and Nanotechnology (ICN2), CSIC and BIST, Campus UAB, Bellaterra, Spain; 7 Centro de Investigación Biomédica en Red Sobre Enfermedades Neurodegenerativas (CIBERNED), Instituto de Salud Carlos III, Madrid, Spain; 8 Red Española de Terapias Avanzadas (RED-TERAV), Instituto de Salud Carlos III, Madrid, Spain; 9 Centro de Investigación Biomédica en Red de Enfermedades Cardiovasculares (CIBERCV), Instituto de Salud Carlos III, Madrid, Spain; 10 Unitat de Neurociència Traslacional, Parc Taulí Hospital Universitari, Institut d’Investigació i Innovació Parc Taulí (I3PT), Sabadell, Spain

**Keywords:** Brain injury, Ischemic stroke, Spreading depression, Cortical spreading depolarization, Neuroprotection, Electrophysiology

## Abstract

Cortical spreading depolarization (CSD) is a pathophysiological event critically implicated in ischemic stroke and other brain disorders. It consists of slowly propagating waves of massive neuronal and glial depolarization in cerebral gray matter, accompanied by spreading depression of cortical activity. CSD disrupts ion homeostasis, alters cerebral blood flow, and contributes to neuronal death in vulnerable tissue. This comprehensive review summarizes both classic and recent studies on CSD mechanisms and their role in brain damage progression after stroke. We also review potential neuroprotective strategies to mitigate CSD-induced damage and discuss available technologies for detecting CSD. Advancing our understanding of CSD mechanisms, combined with targeted neuroprotective strategies and improved monitoring techniques, holds promise for reducing stroke-related brain injury and guiding personalized recovery approaches.

## Introduction

Ischemic stroke is a leading cause of death worldwide and is associated with severe disabilities, which hinder the patient’s quality of life [158]. Currently, endovascular thrombectomy and/or intravenous administration of recombinant tissue-type plasminogen activator (rtPA) remain as the only effective strategies to treat acute ischemic stroke [23]. Nevertheless, only a reduced group of patients can benefit from these treatments due to their narrow therapeutic time window (3–4.5 h and up to 24 h or beyond for endovascular thrombectomy in selected cases) and potential side effects, such as systemic thrombolysis or intracerebral hemorrhage [23, 93]. Therefore, it is of utmost importance to understand the underlying mechanisms promoting ischemic stroke damage expansion to develop new therapeutic avenues.

Injury mechanisms unleashed soon after stroke onset include excitotoxicity, loss of ion homeostasis, inflammatory cytokines, and reactive oxygen species (ROS) production, altogether culminating in cell death [177]. Another important phenomenon that has been identified after stroke is the occurrence of cortical spreading depolarizations (CSD), which are massive depolarization waves of neuronal and glial cells that propagate at a rate of 2–9 mm/min through cerebral grey matter [175]. These waves advance from the core region to healthy brain areas, inducing a marked change in its absolute potential. Originally, these pathophysiological events were perceived as a process occurring in laboratory animals under experimental conditions until they were identified in some human neuropathological diseases by the Cooperative Study on Brain Injury Depolarizations (COSBID) international research consortium [96]. Subsequently, the study of CSD has become an emerging field of research that can guide early diagnosis and prognosis after stroke, facilitating therapeutic interventions [36, 37]. Notably, the parallel improvement of technologies for monitoring brain injury (e.g., electrocorticography) [96] and the research in newly developed advanced materials [106], which allow high-fidelity mapping of CSD, guarantees the continued development of this effervescent field of study.

The purpose of this review is to outline the molecular and cellular mechanisms involved in the appearance and propagation of CSD and their contribution to brain damage expansion after stroke. We discuss potential neuroprotective strategies aimed at mitigating CSD-induced damage, highlighting pharmacological interventions that may improve stroke outcomes. Finally, we describe the currently available technologies to identify and characterize CSD, which can supply bench-to-bedside diagnosis tools for promising personalized and effective stroke recovery treatment.

## Pathophysiology of stroke

Stroke is defined as a brain function disturbance caused by an interruption in blood supply originating from either a blockage or rupturing of a blood vessel [2, 102]. Stroke is a global health-care problem due to its high rate of mortality and adult-induced disability [23, 93]. It is the second leading cause of death worldwide, and the current trends indicate that its prevalence will increase by 2030 [62]. Simultaneously, stroke mortality is expected to diminish due to advances in stroke treatments, though this could represent an increase in the social-care system costs, becoming a big socio-economic problem [48]

Aging is considered the main non-modifiable risk factor for ischemic stroke. Aged stroke patients have a higher risk of mortality and morbidity and poorer functional outcomes than young people. In addition, patient age modifies the influence of sex in ischemic stroke. In early life, the burden of ischemic stroke is higher in men, but stroke becomes more common and debilitating for elderly women [130]. There are also some modifiable risk factors associated with a major probability of suffering a stroke, such as hypertension, smoking, obesity, diet, sedentary life, diabetes, and alcoholism [25]. Stroke can be classified into two basic types, namely ischemic stroke and hemorrhagic stroke. In ischemic stroke, obstruction is produced by thrombus or embolus, representing almost 80–87% of all the cases. With a lower incidence, hemorrhagic stroke results after a brain vessel ruptures and bleeds into the surrounding tissue. The blood then compresses the surrounding brain tissue and activates the inflammatory cascade [178].

Brain cells require a constant blood supply to maintain O_2_ and glucose within the physiological range. However, obstruction of blood vessels leads to a deprivation of these essential components, culminating in reactive oxygen species (ROS) formation, release of glutamate, intracellular Ca^2+^ accumulation, and induction of inflammatory processes [52]. Despite the redundancy of the central nervous system vasculature, such as the circle of Willis, which helps mitigate the consequences of blood flow alterations by reversing flow [33], maintaining adequate perfusion is crucial for improving outcomes in patients with stroke. This preservation of perfusion supports neuronal function and survival [3]. However, in some types of ischemic stroke, it is insufficient to maintain an adequate blood supply. Consequently, prolonged ischemia initiates a cascade of deleterious events that can lead to neuronal death and largely irreversible brain damage [102].

Studies in animal models and clinical research have demonstrated the presence of an *ischemic core* or an area in which cerebral blood flow (CBF) is severely compromised, with irreversible damage [159]. The ischemic surrounding area called *ischemic penumbra* is a region with impaired electrical activity but preserved cellular metabolism, and it is potentially salvageable if treatments are applied in a short time frame window (few hours) from the stroke onset [52]. Therefore, preventing this brain tissue from progressing to irreversible damage is a main objective of stroke therapeutic development [125].

Notwithstanding research efforts and clinical trials performed during the last decades, currently, there are no broadly effective pharmacological treatments against stroke. Reperfusion strategies represent the most effective interventions during the acute phase of primary stroke. Their main goal is the recovery of hypoperfused salvageable tissue [125]. The gold standard in acute ischemic stroke treatment is mechanical thrombectomy, in which a catheter-based device is used to remove blood clots. However, there are some limitations associated with this intervention. In 7 to 19% of cases, there are procedure-related complications such as device fracture, vessel perforation, hemorrhage, and non-target artery embolization [143]. The probability of suffering these complications increases in patients with old age, diabetes mellitus, and uncontrolled hypertension [34]. Besides thrombectomy, recombinant tissue plasminogen activator (r-tPA) is currently the only approved US Food and Drug Administration and European Medicines Agency drug treatment for acute ischemic stroke [123]. R-tPA promotes the enzymatic degradation of blood clots, but the main limiting factor is the relatively short time frame of administration. Thus, the therapeutic benefits decline when r-tPA is administered over 3 to 4.5 h after stroke onset [180]. However, for selected patients, endovascular thrombectomy can be performed up to 24 h after stroke onset, offering an extended treatment window in carefully selected cases [23, 63]. Another limitation of this treatment is the risk of symptomatic intracerebral hemorrhage, reported in 6–7% of cases [169]. Moreover, mechanical and intravenous thrombolysis with r-tPA often fails to recanalize proximal artery occlusions caused by large clots [125]. This “no-reflow” phenomenon is characterized by incomplete microcirculatory restoration after reopening the occluded vessel. Pericyte-induced capillary contraction, driven by ischemia-mediated release of reactive oxygen and nitrogen species (ROS), contributes to this effect [85]. In addition, microvascular thrombosis, endothelial swelling, and inflammatory responses also play major roles. Therefore, even when patients meet the criteria for treatment, reversing stroke-induced neurological deficits depends on more than just clot removal. Overall, available evidence highlights the importance of timely detection of symptom onset and the use of reperfusion strategies to minimize secondary brain damage [125].

Spreading depolarizations (SD) were first described by the Brazilian neurophysiologist Aristides A. P. Leão in 1944, while he was studying epilepsy seizure activity in the rabbit cortex. In those experiments, both injurious and non-injurious focal electrical or mechanical stimuli led to the development of a wave that subsequently progressed to electrocorticographic silence, which slowly propagated across the cortex [97]. Following their discovery, research on these waves has gained importance. Technological advances later demonstrated their occurrence in several human diseases, including epilepsy, traumatic brain injury, and stroke [96]. This cortical slowly propagation wave was termed CSD and is now considered both a diagnostic marker and a therapeutic target in several neurovascular diseases, including stroke [10].

## Spreading depolarization: Physiological and pathological responses

### Breakdown of ion homeostasis and glutamate excitotoxicity

Spreading depolarizations are described as an abrupt and sustained mass depolarization of neuronal and glial cells that propagates at velocities of 1.7–9.2 mm/min across the cerebral grey matter [58, 68, 175]. The underlying mechanisms leading to the onset of this phenomenon are not completely understood, although it is known that increased extracellular levels of K^+^ and increased excitatory amino acids, mostly glutamate, are involved in SD onset [155]. Progression of SD produces a near-complete breakdown of ion homeostasis, functional and structural alterations of the cerebral vasculature, and depression of electrical activity, leading to neuronal swelling and distortion of dendritic spines [90].

**Fig. 1:**
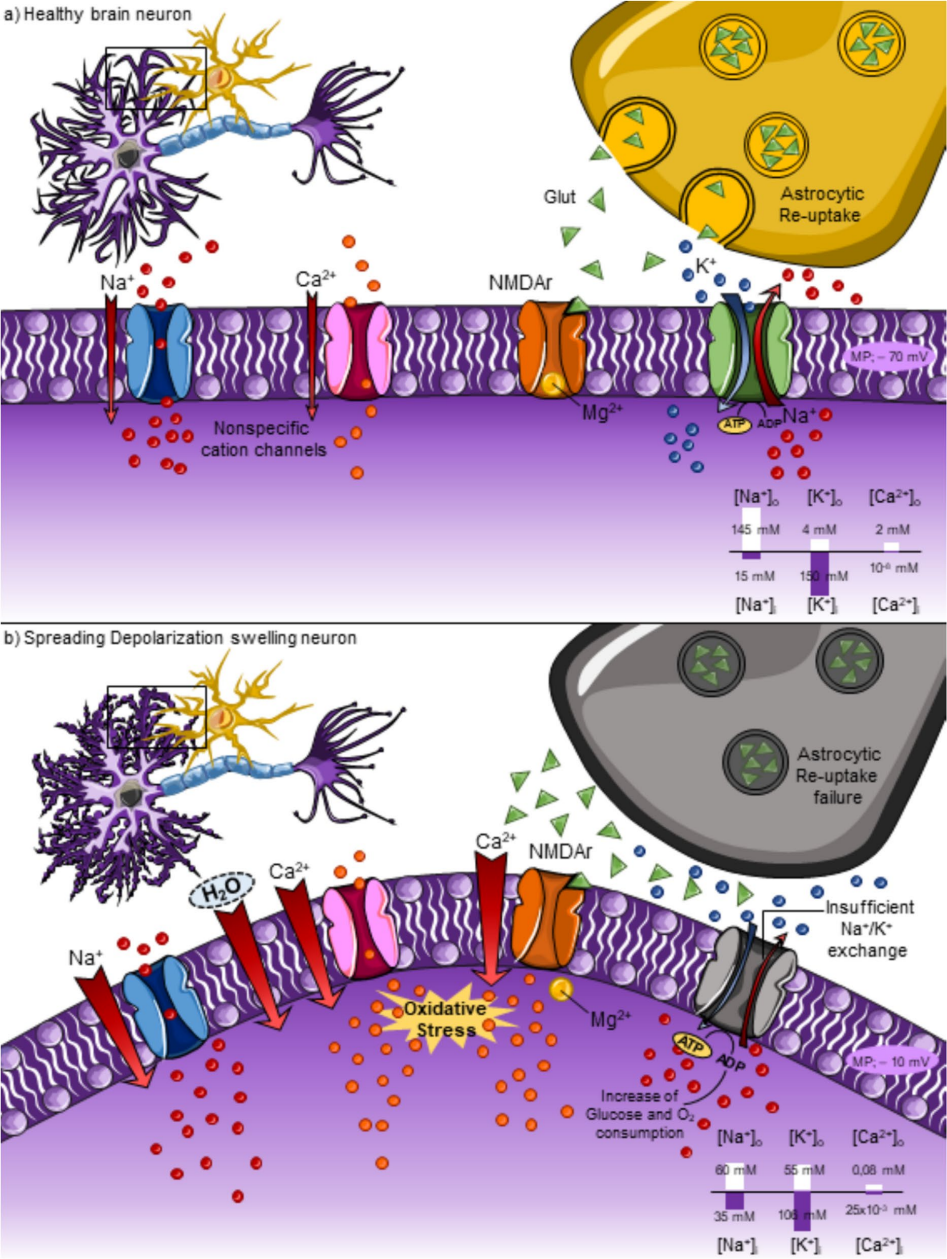
Pathophysiological mechanisms of spreading depolarization. **a** In a physiological situation, the cationic inward current mediated by Na^+^ and Ca^2+^ channels is balanced by the Na^+^/K^+^ ATP-dependent pumps ensuring the double *Gibbs-Donnan* equilibrium characterized by iso-osmolarity across the plasmatic membrane. In addition, the NMDA channel is blocked by Mg^2+^ inhibiting the entry of Ca^2+^ from the extracellular space. The susceptibility of these channels to glutamate is reduced due to the astrocytic-mediated clearance of this excitatory aminoacid from the extracellular space, reducing also the excitotoxicity probability. **b** In contrast, in pathological conditions, the cationic exchange mediated by Na^+^/K^+^ pumps is insufficient to face the massive inward current increases. Therefore, a near-complete sustained depolarization is induced negatively shifting the membrane potential (MP) from −70 to −10 approximately. Disturbance of homeostasis promotes intracellular hyperosmolarity, causing water entry, neuronal swelling, and edema formation. Depolarization also removes the Mg^2+^ block of NMDA receptors, increasing their susceptibility to glutamate, especially when astrocytic reuptake fails. As a result, Ca^2+^ accumulation triggers the production of highly toxic reactive oxygen and nitrogen species, ultimately leading to neuronal death

In a non-pathological situation (Fig. 1a), Na^+^ and Ca^2+^ entry produce a small dendritic inward cation current [80, 89]. There is an outward current to compensate for this entry achieved by Na^+^/K^+^ ATP-dependent pumps establishing the double Gibbs-Donnan equilibrium characterized by iso-osmolality across the membrane [95]. Levels of excitatory amino acids such as glutamate are regulated by excitatory amino acid transporters (EAAT)-mediated re-uptake in astrocytes, an effect that prevents extracellular-induced excitotoxicity. This rapid clearance process limits the interaction of glutamate with neuronal NMDA receptors (NMDAr), reducing the possibility of cationic influx through these channels [149].

In contrast, under conditions of pronounced metabolic compromise, such as ischemia (Fig. 1b), the energy demand mismatch impairs Na^+^/K^+^-ATPase activity, an effect followed by disruption of ionic gradient maintenance, leading to a massive influx of Na^+^ and Ca^2+^ through specific ion channels, such as voltage-gated Na^+^ channels and NMDA receptors. This results in the progression of sustained membrane depolarization [10]. The subsequent intracellular hyper-osmolarity contributes to H_2_O entry, leading to neuronal swelling and distortion of dendritic spine structure, resulting in edema formation, which increases intracranial pressure [153]. Depolarization of neurons removes the Mg^2+^ block from NMDA receptors, making them more sensitive to fluctuating glutamate levels. Sustained overactivation increases Ca^2+^ influx and other cations, which triggers superoxide and nitric oxide production, ultimately leading to neuronal death. Furthermore, these two reactive molecules can combine with each other to form peroxynitrite, a highly toxic anion [15, 124]. Reactive oxygen molecules also play a role in the early expression of inflammatory mediators, such as adhesion molecules and cytokines, enhancing the accumulation of leukocytes in the ischemic regions [1]. This aggregation leads to thrombosis, decreases cerebral perfusion, and damages the blood-brain barrier (BBB). More specifically, BBB function is compromised through dual mechanisms: (i) increased expression of matrix metalloproteinase-9 (MMP-9) in neurons and astrocytes, which degrades basal lamina components, and (ii) activation of caveolin-1–mediated transcytosis in endothelial cells, resulting in increased vesicular transport across the BBB without overt tight junction disruption [64, 131]. In addition, ROS activate the p53 signaling pathway and caspase-dependent programmed neuronal death [82].

Moreover, it is described that cytosolic Ca^2+^ accumulation enhances the release of glutamate from synaptic vesicles to the extracellular space [185]. Furthermore, glutamate astrocytic transporters, in a scenario of ATP depletion, collapse, and this results not only in the failure of glutamate re-uptake but also in its efflux via uptake reversal [190]. There are other neurotransmitters and neuromodulators released during SD due to the disruption of ionic homeostasis, such as aspartate, glycine, GABA, catecholamines, adenosine, and ascorbate. In fact, these are, together with the increased extracellular levels of K^+^, the potential candidates responsible for anoxic depolarization propagation across nearby neurons from the ischemic core region to the penumbra [10].

Neurons have several mechanisms to cope with this pathological state and restore homeostasis, but these mechanisms rely heavily on ATP consumption. In energy-compromised regions such as the ischemic core, ATP availability is insufficient, delaying recovery and worsening injury [10]. In metabolically compromised but partially preserved regions (i.e., penumbra), ATP consumption increases to sustain Na^+^/K^+^-ATPase activity, resulting in a 30–60% decrease in tissue glucose and a shift from aerobic to anaerobic metabolism, with accumulation of lactate and CO_2_. Thus, there is a shift of extracellular pH from alkaline towards acidic, potentiating neuronal damage beyond classical excitotoxicity [10, 50]. Consequently, these metabolic alterations that end in acidosis are thought to exacerbate ischemic brain injury by causing protein denaturation, activation of acid-sensing ion channels, and release of ferrous ion [184].

### Cerebral hemodynamic responses to spreading depolarization

Neural activity both affects and is affected by changes in CBF, a concept well known as *neurovascular coupling* [59]. Neural activity increases CBF to meet metabolic demands in a process called *functional hyperemia*, which is mediated by numerous factors such as ions, neurotransmitters, and other vasoactive molecules. Specifically, when energy demand is increased by the activation of some particular brain regions, local O_2_ and glucose levels decrease [6]. In response to that, there is a release of K^+^ from astrocytic endfeet, which triggers the activation of large-conductance Ca^2+^-activated potassium (BK) channels. The rise in extracellular K^+^ concentrations hyperpolarizes vascular smooth muscle cells (VSMCs), leading to vasodilation. This hyperemia ensures that enough O_2_ and nutrients reach metabolically demanding neuronal areas [86, 108].

**Fig. 2:**
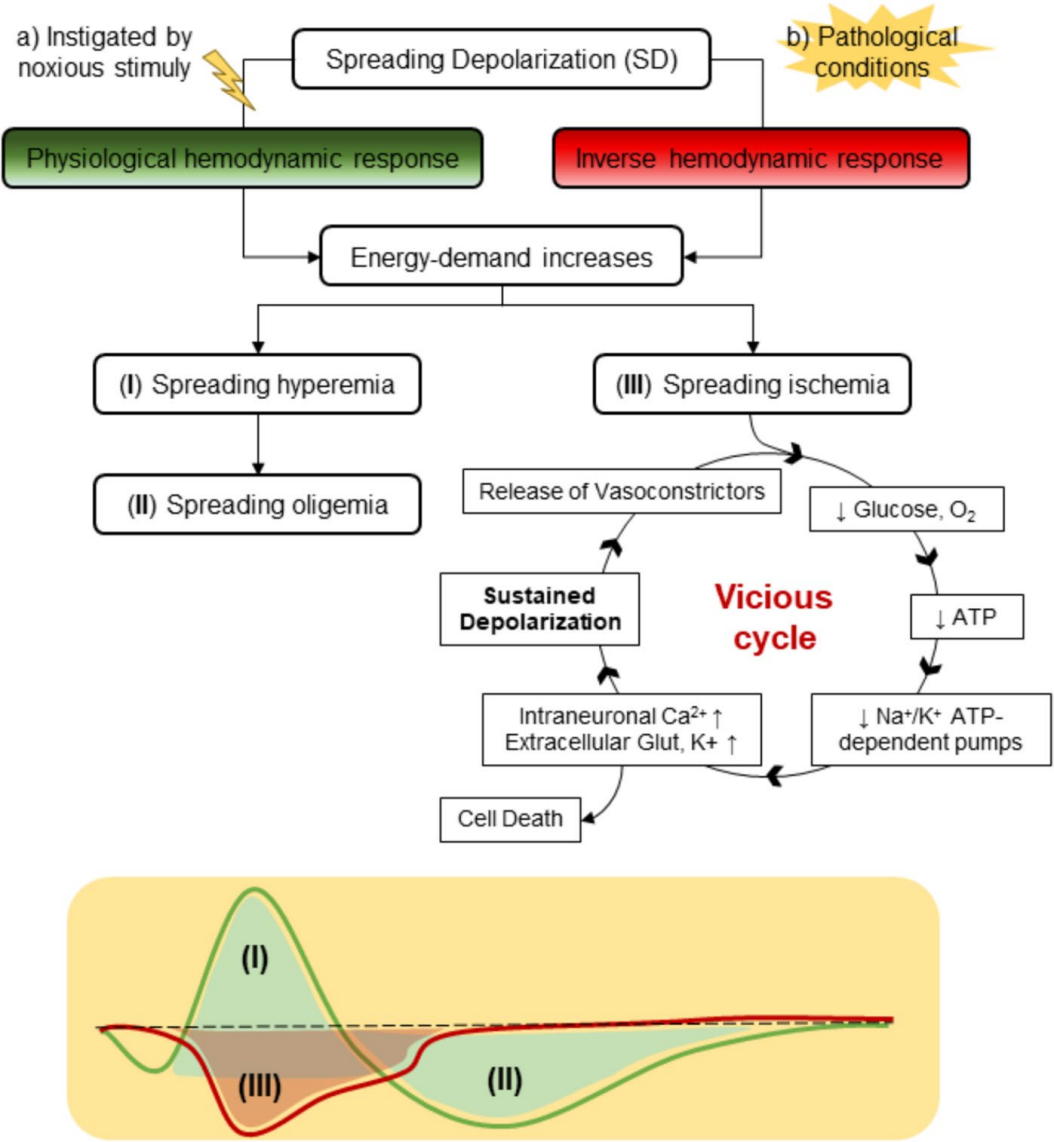
Hemodynamic changes mediated by the neurovascular unit in response to spreading depolarization. **a** Spreading depolarization waves instigated in a non-pathological state induce hemodynamic changes like those mediated by the neurovascular unit during physiological brain activation. Thus, the energy demand necessary to fuel the Na^+^/K^+^ pumps leads to an increase of cerebral blood flow (CBF), a process called spreading hyperemia (I). Normally, this transient alteration is followed by a decrease of CBF below the baseline, namely spreading oligemia (II), to clear the vasoactive mediators released in the first peak. **b** In contrast, in pathological conditions, neurovascular coupling induces reversion of these changes. Thus, there is a reduction of CBF, known as spreading ischemia (III). This scenario is maintained over time in a vicious cycle in which the constant release of vasoconstrictors and the lack of energy substrates to balance the ionic disturbances evolve into cellular death

Spreading depolarizations can be induced by noxious stimuli such as mechanical damage, electrical stimulation, or chemical agents (e.g., concentrated KCl solutions). In these cases, SDs reproduce the physiological hemodynamic response mediated by neurovascular coupling [36]. Specifically, regional CBF rises above baseline in affected cortical regions for at least 2 min, a process termed spreading hyperemia (Fig. 2a(I)). This phenomenon provides the overactivated tissue with enough oxidative substrates to restore homeostasis, activating the energy-dependent Na^+^/K^+^ pumps. Following this initial hyperemic response, there is a decrease or even a sustained suppression of rCBF known as *spreading oligemia* (Fig. 2a(II)). In this state, rCBF remains 10–40% below baseline for an hour or longer in order to transiently attenuate vascular responses to dilatory mediators released by the energy-compromised tissue [24].

Neurovascular coupling is normally tightly regulated to maintain homeostasis. However, under pathological conditions such as ischemic stroke, subarachnoid hemorrhage, or traumatic brain injury, this regulation can become inverted. Instead of vasodilation, the propagation of neuronal and glial depolarization produces paradoxical vasoconstriction (hypoperfusion). This phenomenon is called *spreading ischemia* (Fig. 2a(III)) and results in reduced CBF, hindering the arrival of O_2_ and glucose to the affected areas and creating a vicious cycle that exacerbates neuronal damage [86, 108].

Although the precise mechanisms underlying this inverted neurovascular response remain incompletely understood, several contributing factors have been identified. Elevated intracellular Ca^2+^ levels in astrocytes can enhance the activation of BK channels, increasing K^+^ release into the perivascular space. When extracellular K^+^ concentrations exceed a certain threshold, VSMCs become depolarized, leading to their contraction and subsequent vasoconstriction. In addition, the release of vasoconstrictors [10] and decreased nitric oxide availability (due to scavenging by ROS or inhibition of nitric oxide synthases’ expression/activity) [38, 136] further contribute to this pathological response [7, 36]. Due to this persistent vasoconstriction, the activation of energy-dependent membrane pumps, such as the Na^+^/K^+^-ATPase, is impaired, which is essential for restoring ionic gradients. As a result, the failure to repolarize cells sustains neuronal depolarization, cytotoxic edema, and toxic intracellular ion accumulation, thereby increasing the risk of irreversible injury [36].

Several vasoconstrictor mediators have been implicated in spreading ischemia. Their effects vary depending on the type of brain vascular bed (e.g., pial versus parenchymal arterioles and capillaries) and the species studied, demonstrating the complexity of these responses [10]. These mediators include perivascular K^+^ ([12], arachidonic acid metabolites such as lipoxygenase-derived 20-hydroxyeicosatetraenoic acid [53], and cyclooxygenase-derived prostanoids, including prostaglandin F_2α_ [139, 140] and thromboxane A_2_ [57]. However, the contribution of other vasoconstrictors, such as endothelin [10, 60], serotonin, norepinephrine, and adenosine, remains controversial [10]. Notably, recent studies have identified additional factors that may influence vascular responses during SDs. For instance, the activation of pannexin-1 channels and the nucleotide-binding and oligomerization domain-like receptor family pyrin domain-containing 3 inflammasome in neural cells can lead to the release of pro-inflammatory cytokines like Interleukin-1 beta (IL-1β), which may indirectly exacerbate vasoconstriction through increased generation of ROS leading to nitric oxide scavenging, thereby contributing to spreading ischemia [5, 144]. Furthermore, the role of high mobility group box 1 protein has been implicated in neuroinflammation and could also indirectly influence vascular tone during SDs [144]. These findings underscore the complexity of vascular responses during SDs and indicate that additional studies are needed to clarify the underlying mechanisms.

### Cytotoxic and vasogenic edema triggered by spreading depolarizations

Spreading depolarizations and brain edema are tightly interconnected processes that mutually reinforce each other during acute brain injury. SDs represent the cellular mechanism underlying cytotoxic edema in the grey matter: the near-complete collapse of ionic gradients across neuronal and glial membranes causes a massive influx of Na^+^, Ca^2+^, and Cl^-^, accompanied by osmotic water entry and cellular swelling. This process, initially reversible, becomes deleterious when SDs recur or persist under conditions of metabolic compromise. As summarized by Dreier et al. in their comprehensive review [39], cytotoxic edema induced by SDs has been consistently demonstrated in experimental models of ischemia and anoxia [129, 150, 164] and correlates with diffusion-weighted magnetic resonance imaging (MRI) changes during stroke [112]. Dreier and colleagues proposed that SD-related cytotoxic edema is not a secondary phenomenon but a central mechanism of neuronal injury in both ischemic and hemorrhagic stroke. Moreover, the associated microvascular constriction, spreading ischemia, can propagate perfusion deficits into surrounding tissue, thereby amplifying cytotoxic edema and promoting irreversible cell death [39, 42].

Subsequent experimental work by Menyhárt et al. revealed that acute astrocyte swelling during ischemia impairs glutamate uptake, leading to toxic extracellular glutamate accumulation and the emergence of large-scale, simultaneous depolarizations [108]. These events represent an amplified form of SD that recruits extensive cortical territories and triggers edema-associated neuronal death. Pharmacological or osmotic interventions that reduce astrocytic swelling, such as aquaporin-4 blockade or mannitol administration, effectively prevented the occurrence of simultaneous depolarizations, underscoring the central role of astroglial edema in sustaining SD propagation. Finally, it was demonstrated that brief intracranial pressure spikes, secondary to edema, even of mild amplitude, can trigger SDs in the ischemic penumbra by acutely worsening cerebral perfusion and oxygenation [118]. Together, these findings delineate a vicious cycle in which SDs promote cytotoxic and vasogenic edema, while mechanical or osmotic stressors further increase tissue susceptibility to SD generation. This reciprocal interaction between SDs and edema is likely to contribute to the rapid deterioration observed in malignant hemispheric stroke.

## Cortical spreading depolarizations: Induction mechanisms and pharmacological interventions

### Experimental induction of CSDS

Cortical spreading depolarizations were described for the first time by the neurophysiologist Aristides A. P. Leão [97]. With the aim of studying experimental epilepsy, Leão observed that repetitive electrical stimulation of the cortex of a rabbit depressed electrical activity towards this brain area. Using electrocorticography (ECoG), it was shown that this phenomenon spreads slowly across the cortical surface at approximately 3 mm/min. Specifically, the depression was characterized by a transient loss of neuronal excitability, which returned to baseline levels after a short period. Leão termed this phenomenon “spreading depression,” and it has since become a fundamental concept in neurophysiology, particularly in the study of migraine aura and various neurological disorders [97, 157].

To better understand the propagation of this event, in vivo and in vitro models have been developed over the years. At the in vitro level, CSD has been extensively studied in chicken and amphibian retina, as well as in cortical, hippocampal, and cerebellar brain slices, and neuronal cultures [87, 91, 109, 119]. These in vitro models offer some advantages over in vivo approaches and have significantly contributed to understanding the fundamental properties of CSD. The simplified experimental environment in which these studies are carried out enables better control of physiological variables such as temperature, oxygenation, and pH, thereby minimizing confounding factors. Moreover, these approaches have facilitated the assessment of CSD propagation in specific brain regions (e.g., cortex and hippocampus) and facilitate the evaluation of the effects of various pharmacological compounds on the dynamics of CSDs [10, 49].

Multiple methods have been described to trigger CSD, being the most used the direct application of KCl, electrical stimulation, or induction of trauma. Administration of increasing concentrations of KCl [11] or increasing volumes of KCl at fixed concentrations [56] has been used to determine the threshold concentrations or the minimal critical volume to trigger CSD. On the other hand, single pulse electrical stimulations or high frequency trains through bipolar or monopolar needles or cortical cup electrodes are considered suitable methods to determine the CSD threshold. However, threshold reproducibility is difficult to achieve due to variability in impedance, electrode shape, and brain surface properties [56]. Lastly, the trauma model is induced with a needle or by exerting mechanical pressure to the tissue. This triggering method reduces the sensibility to determine gradually the threshold to induce CSD, generally developing an all-or-none response [8]. Recently, less-invasive CSD induction methods based on optogenetics have been reported [77] and demonstrated to allow for a reliable and reproducible on-demand induction of CSD [30]. However, it should be noted that the mentioned triggering mechanisms generate transient depolarizations. To study depolarizations linked to pathologies that metabolically compromise brain activity (i.e., ischemic stroke), which are generally sustained over minutes or occur in simultaneous waves, more indirect methods such as arterial clamping or photothrombotic stroke models are better suited [107].

### Neuroprotective interventions targeting CSDs

Continuing studies conducted both in vivo* and *in vitro [87, 91, 109, 119] have well established the close relationship between CSD and neurological diseases, highlighting the need to counteract the onset and spread of this secondary brain-damaging phenomenon (Lauritzen et al., 2010). Thereby, numerous experimental studies tried to target these waves closely linked to damage propagation towards the penumbra area by different neuroprotective avenues. However, effective translation into clinical practice is still in its infancy [37, 84]. An ideal treatment should, obviously, target multiple altered pathophysiological mechanisms. However, disruptions in energy supply after an insult hinder the therapeutic modulation of various affected mechanisms simultaneously. As a result, several individual strategies have been proposed to counteract CSD at different stages of its progression, such as blocking initiation, modulating propagation, reducing wave amplitude, reducing hemodynamic response, and reversing the inverse response. The main targets for these interventions are NMDA, GABA, AMPA, or opioid receptors, among others. Ketamine is one of the most recognized and effective substances in clinical settings for counteracting CSDs, while valproate has shown potential but remains less established in this context [84].

More in detail, NMDA receptor antagonists are key targets to inhibit CSD onset and expansion due to their role in exacerbating ionic imbalance and excitotoxicity. Activation of these receptors under physiological conditions promotes an increase in extracellular K^+^ concentration, simultaneously with the influx of Ca^2+^ and Na^+^, leading to the release of excitatory neurotransmitters (e.g., glutamate) [94]. Antagonists such as ketamine and MK-801 have been experimentally shown to prevent CSD onset and expansion. Specifically, ketamine acts as a non-competitive NMDA antagonist, reducing Ca^2+^ influx, glutamate release, microglial inflammation, and promoting anti-apoptotic protein expression like B-cell lymphoma 2 (Bcl-2) [104, 113, 120, 142, 187]. Human studies have also shown that ketamine significantly reduces the frequency of CSD events in patients with acute brain injury [72] (Table 1).

**Table 1:** Summary of pharmacological targets and mechanisms involved in cortical spreading depolarization (CSD) inhibition. The table outlines three different therapeutic approaches (NMDA receptor antagonist, anesthetics, anti-migraine drugs) that intervene in the CSD cascade (onset, expansion, susceptibility, appearance, and propagation), proposed mechanisms of action, and examples of promising drugs

Target	Inhibit	Mechanism of action	Promising drugs	References
NMDA receptor antagonist	Onset and expansion	Decrease the excitotoxicity mediated by neurotransmitter release and avoid the cationic influx	Ketamine	[113] [120] [187] [142] [104] [72]
			MK-801	
Anesthetics	Susceptibility and appearance	Mechanism still unknown, partial antagonism of NMDA receptors	Sevoflurane	[83] [181]
			Isoflurane	
Anti-migraine drugs	Appearance and propagation (affect the hemodynamic response)	Anticonvulsant during epileptic seizures modulating GABAA receptors	Topiramate	[9] [173] [128]
			Flunarizine	

Inhaled anesthetics modulate susceptibility and initiation of CSDs, likely through partial antagonism of NMDA receptors [182]. Among these, sevoflurane has shown the greatest potential by reducing the frequency of CSDs in a dose-dependent manner and exerting neuroprotective effects mediated via the Toll-like receptor 4 and nuclear factor-kappa B (TLR4-NF-κB) signaling pathway. In contrast, although isoflurane has also been evaluated, its use is associated with adverse effects such as neurodegeneration and increased amyloid beta protein levels [181] (Table 1).

Similarly, anti-migraine drugs such as topiramate and flunarizine have demonstrated efficacy in reducing CSD occurrence and propagation, supporting the established link between CSD and migraine aura pathophysiology [8, 95]. Topiramate, a GABAA receptor modulator, decreases CSD frequency in a dose-dependent manner [9], while flunarizine, a Ca^2+^ channel blocker, mainly affects the hemodynamic response [128, 173]. In addition, vagus nerve stimulation, a treatment used for migraine, also shows inhibitory effects on CSD in animal models [29].

Together, these findings support a personalized therapeutic approach that modulates distinct properties of CSD to provide neuroprotection in neurological disorders. Nonetheless, to effectively apply such treatments clinically, improved neuromonitoring methods are needed to identify specific pathophysiological alterations in each patient.

## CSDs in stroke

### Identification of CSDs in experimental models of stroke

It was not until decades later when SD was first observed in animal models of focal ischemia. Specifically, through the use of ion-selective microelectrodes, it was possible to record a large increase in extracellular potassium concentration in rat brain cortex [21, 168].

Middle cerebral artery occlusion has been described as the gold standard model to mimic ischemic stroke-induced brain damage in experimental conditions. Occlusion of this artery leads to an enlargement of the lesion and cytotoxic edema, processes strongly associated with the occurrence of SDs that cyclically propagate around the injured site [88, 114, 186]. The most malignant variant of SDs arises in the ischemic core, where the reversible depolarization component transitions into a sustained negative ultraslow potential, an irreversible event termed terminal SD. This transformation occurs within less than 5 min of the ischemic onset and marks the beginning of the cellular death cascade [122].

In the more severely affected core-surrounding tissue, where neuronal activity is completely suppressed, SDs can still propagate but are unable to induce further changes in electrical activity. Therefore, these events are known as isoelectric SD, and they are distinct from those occurring in the tissue with residual activity. These slow propagating waves, so-called peri-infarct depolarizations (PID), originate in the ischemic core and propagate to the metabolically active penumbra area, being considered a causative factor in the progression of brain damage. Notwithstanding, what all the SD variations have in common is that under certain cerebral pathological conditions, they serve as an immediate biomarker of escalating brain damage [70].

Despite strong experimental evidence obtained in animal models, SDs were long thought to occur exclusively under laboratory conditions and not in human disease. This misconception was largely due to the technical limitation of earlier detection systems, such as scalp EEG recordings, which lacked the sensitivity to capture these slow and spatially restricted events [134, 155].

### Clinical detection and characterization of CSDs

The presence of SD in stroke patients was corroborated with the appearance of the *Cooperative Studies on Brain Injury Depolarizations* (i.e., COSBID) international consortium [49, 148]. In these pioneering studies, PIDs were monitored for the first time in 26 brain-injured patients that suffered from traumatic or spontaneous intracranial hematomas and/or subarachnoid hemorrhage requiring craniotomy, placing ECoG recording strips on the accessible cortex [49, 148]. Strong et al. were the first to record periods of depressed cortical activity that spread at a velocity of 0.6 to 5.0 mm/min, a characteristic feature of cortical spreading depression (CSD). Afterward, Fabricius et al. reported that half of the 12 patients in their cohort experienced a total of 73 spontaneous episodes of spreading depression observed in their ECoG recordings. Specifically, CSDs were detected in 80% of patients with traumatic brain injury (TBI) and 28% of those with spontaneous hemorrhage [49].

Lately, research by Dohmen et al. [35] first highlighted the significance of CSD in malignant hemispheric stroke (MHS) patients, observing their enhanced incidence compared to patients with spontaneous or traumatic intracerebral hemorrhage, as well as subarachnoid hemorrhage [49, 148]. This difference was attributed to the extent of the ischemic penumbra, which is broad after stroke but appears confined to a reduced territory near the structural lesion, and may even be unclear in other types of injuries [19, 35, 73, 167]. This study monitored 16 patients with large MCA infarction during 109 h after injury, recording a total of 127 episodes of CSDs and 42 PIDs, observing a time delay between the onset of monitoring and the first depolarization event of 0.8 to 62.4 h [35]. They found that CSD appeared in clusters, and the duration of ECoG recovery after each CSD tended to increase over time, indicating a progressive deterioration of the metabolic or hemodynamic status, with increasing difficulty in restoring ion gradients and functional activity.

Furthermore, in MHS patients, in addition to ECoG, changes in CBF closely linked to CSD appearance were recorded using laser speckle technology, and, simultaneously, infarct progression was assessed through (MRI) [175]. In this study, 19 CBF changes characteristic of CSD were observed in 7 of 20 patients (13 of them manifested hyperemia, 2 a biphasic response, and 4 decreased blood flow). Moreover, in 70% of patients, a mean of 56 ± 82 CSDs were recorded, accompanied by 30 ± 13 cm^3^ mean infarct progression. Early studies in patients suggested that the appearance and propagation of CSDs in the penumbra are correlated with lesion enlargement [35, 49, 148, 175]. Since then, thanks to advances in technology, CSD is no longer considered an epiphenomenon observed only in animals [155].

### Hemodynamic and metabolic responses to CSDs in stroke

Generally, in both ischemic core and penumbra regions, SDs elicited by brain ischemia lead to dramatic changes in CBF, promoting the transition from a normal vascular response to its opposite. The predominant vasoconstriction exacerbates the preexisting reduction of blood supply to the brain, a fact that has been observed in several animal models such as subarachnoid hemorrhage, hypotension and hypoxia, and middle cerebral artery occlusion [74]. The greater the inversion of hemodynamic responses to SD, the more severe the condition of the tissue becomes [17]. Although SDs can induce a switch to an inverse vasoconstrictive response, the changes in CBF following ischemia are highly heterogeneous and depend on the metabolic state of the tissue, as well as the integrity of the neurovascular unit. Therefore, it is essential to consider that SDs are generally accompanied by spreading hyperemia, which serves to restore both blood supply to the affected brain region and ionic homeostasis. In this way, Binder et al. [17] identified two distinct vascular responses of ischemic CSD in mice after MCA intraluminal thrombin injection: penumbral spreading hyperemia (PSH) and full hemisphere spreading hyperemia (FHSH). The hyperemia that spread uniquely in the penumbra region was followed by severe hypoperfusion, while there was almost no hypoperfusion after FHSH. For that reason, PSHs have been linked to harmful effects and progressive tissue injury, whereas FHSHs have been associated with improved infarct outcomes and the potential preservation of the viability of the infarct core. It was proposed that FHSHs may serve as a biological indicator of vascular tissue recovery. This implies that regions of the cortical tissue within the infarct core remain at least partially viable since necrotic regions are unable to generate spreading hyperemia. The hypothesis is that those episodes of hemispheric hyperemia might contribute to tissue repair by reducing acidosis. Accordingly, targeting the hemodynamic alterations triggered by CSD could be a promising strategy for stroke treatment [17].

Heterogeneity is observed not only in hemodynamic responses but also in metabolic status. Using glutamate-sensitive microelectrodes, Hinzman et al. [74] showed that spreading depression is consistently accompanied by increased extracellular glutamate. This was true in both naïve brains (SDs induced by KCl) and ischemic tissue (embolic focal ischemia model). In naïve tissue, blocking glutamate uptake caused both the direct current (DC) shift during SD and the increase in glutamate levels to last longer. Recovery from both SD and glutamate accumulation requires activation of membrane pumps that depend on ATP. However, due to the mismatch between energy demand and supply following blood flow deprivation, the function of these pumps is impaired, explaining the prolonged duration of both depolarization and glutamate excess [74].

Importantly, the close relationship between CSD outcomes and tissular affection in terms of metabolism and neurovascular responses was also observed in patients [28]. In MHS patients, the incidence of SDs increases under two conditions: when metabolic impairments are present and when neurovascular coupling is inverted in the peri-infarct region [35].

For instance, in TBI and MHS patients, SDs’ appearance and propagation are closely linked with reduced glucose and pyruvate concentrations, alongside elevated glutamate and lactate levels in the extracellular space. Thus, the length of the activity suppression triggered by SDs corresponds to the period during which there is an imbalance between energy supply and demand, reflecting the extent of the metabolic disturbance [122, 175]. Based on that, the lactate/glucose ratio is useful to determine unfavorable outcomes in patients when it is increased, as happened in the work of Goodman et al. [61] in patients with TBI [61, 160].

Secondary ischemic lesions (e.g., delayed infarct progression) and unfavorable outcomes were observed in MHS, TBI, and ischemic hemorrhage (IH) patients despite decompressive craniectomy, and these findings were correlated with SD detection [88, 122]. Notably, variations in glutamate, glucose, lactate, and pyruvate levels were measured in the tissue surrounding the infarct core, showing a strong association with infarct progression [122, 175]. Rapid-sampling microdialysis and enzyme-based microelectrodes are the preferred techniques to determine the concentrations of these metabolites [122]. Specifically, in these studies, two microdialysis probes were inserted into the peri-infarct region, at different distances from the ischemic core, to assess whether the changes in these metabolites followed a gradient toward the penumbra. In patients with infarct progression, glucose and pyruvate levels were reduced, whereas glutamate levels and the lactate/pyruvate ratio were increased compared to patients without this condition, particularly during the first hours after the surgical procedure. Notably, changes were observed mostly with the microdialysis probe placed further away from the infarct border. Interestingly, all the monitored metabolites were also altered at shorter distances [122, 176].

To assess the complex pathophysiological alterations following MHS in the area surrounding the infarction, ECoG is better than standard sampling using microdialysis probes. Notwithstanding, the rise in glutamate levels manifested in the first hours after the stroke may be useful as a biomarker of secondary damage [122].

### Physiological variables influencing CSDs in stroke

The alterations in metabolite concentrations are not isolated factors influencing the onset and progression of SDs. Other physiological variables are also closely associated with poor outcomes when coupled with SDs [138]. In this context, Schumm et al. [138] demonstrated that decreases in mean arterial pressure (MAP) and cerebral perfusion pressure (CPP), along with increases in intracranial pressure (ICP), preceded the occurrence of 1692 SDs in at least 60 patients with malignant hemispheric stroke (MHS). In the same study, they experimentally evaluated the roles of blood pressure and temperature in a mouse model of middle cerebral artery occlusion. Their findings indicated that hypotension (i.e., low blood pressure) is a major contributor to the inverse hemodynamic response and the hypoperfusion observed following KCl-induced SDs, which in turn prolongs their duration. Conversely, hypothermia appeared to exert a neuroprotective effect by promoting hyperemia (i.e., increased blood flow) in response to elicited SDs [138].

The interplay between various physiological variables and the increased risk of SDs were further explored by Chau et al. [28]. In their study, they monitored multiple parameters such as MAP, heart rate, diastolic and systolic blood pressure (DBP), end-tidal CO_2_ (ETCO_2_), and peripheral O_2_ saturation (SpO_2_), among others, in ten patients with MHS following large territory infarctions. A platinum cortical electrode was implanted in the accessible peri-infarct region after decompressive hemicraniectomy. SDs were recorded in only five of the ten patients, specifically those in whom the electrode was placed in viable peri-infarct tissue. In the remaining five patients, the electrode was positioned within the infarct core, an area lacking detectable electrical activity, limiting the ability to record SDs [28].

Consistent with the findings of Schumm et al. [138], a decrease in MAP, along with reductions in DBP and ETCO_2_, was closely associated with an increased incidence of SDs. Additionally, this study highlighted hyperthermia and elevated white blood cell count as key contributors to SD initiation. On the other hand, certain protective factors were identified: elevated levels of ETCO_2_ and arterial CO_2_ (PaCO_2_) appeared to mitigate the occurrence of these slowly propagating waves [28].

Recently, it has been described how variations in partial tissue O_2_ pressure (ptiO_2_), when coupled with SDs, can serve as a valuable bedside parameter for predicting worse functional outcomes in patients with malignant hemispheric stroke [70]. To avoid placing electrodes within the electrically inactive ischemic core, where SDs cannot be detected, as previously reported by Chau et al. [28] and Dohmen et al. [35], the boundaries of this area were identified using laser speckle imaging. In addition to ECoG electrodes, which were placed 10–15 mm from the infarcted region, a brain tissue O_2_ monitoring probe was also inserted nearby, beneath the pia mater [70].

It is well established that, under experimental conditions, SDs increase the metabolic rate of O_2_ consumption, accompanied by alterations in CBF. Specifically, when neurovascular coupling remains intact, the increase in CBF supports the restoration of ion homeostasis without causing damage to brain tissue. However, when O_2_ consumption becomes excessively high (i.e., as occurs following an ischemic insult), resulting tissue hypoxia leads to prolonged SD duration and extended depression of spontaneous brain activity, thereby exacerbating infarct progression [43, 70]. The biphasic response is the most common, indicating that the neurovascular response is still preserved as well as a relatively healthy metabolic state in the at-risk but still viable peri-infarct tissue. The second most common pattern is the hypoxic response, which likely reflects more severely compromised tissue. However, contrary to expectations, it has not been associated with clustered or isoelectric SDs. To note, a greater occurrence rate and larger magnitude of any of the mentioned ptiO_2_ fluctuations linked to SDs were correlated with improved functional recovery at 6 months. This supports the idea that ptiO_2_ changes in response to SDs reflect healthier, metabolically preserved cortical tissue [70].

Overall, both the inverse neurovascular response (i.e., glutamate excitotoxicity) and the metabolic burden are major contributors to secondary injury following ischemic stroke, cytotoxic edema, and, ultimately, cell death. Thus, there is growing interest in monitoring various bedside variables as complementary information to electrocorticographic recordings in patients suffering from brain injury [70].

### Translational therapeutic interventions targeting CSDs in stroke

Significant efforts have been made not only to understand the underlying mechanisms associated with the appearance and propagation of SDs in the brain after injury [36] and to monitor their progression through invasive and less invasive bedside techniques [67, 68], but also to develop pharmacological approaches aimed at reducing their onset to prevent activity depression and cell death [84]. Hence, the ability to accurately record CSDs shortly after their onset, in addition to recognizing and targeting crucial molecular routes that modulate the human brain’s vulnerability to CSD onset, could be a useful tool to ameliorate stroke and other patients’ outcomes suffering from brain pathologies [84, 96].

The NMDA receptor antagonist ketamine is among the most extensively studied and is currently considered one of the most effective pharmacological agents for reducing the onset and progression of SDs under certain brain injuries, such as TBI, intracerebral hemorrhage (ICH), or after MHS [72, 132].

Several years ago, high-dose enantiomer S(+)-ketamine was shown to improve neurological deficits after a period of common carotid artery unilateral ligation under hemorrhagic hypotensive conditions (i.e., incomplete cerebral ischemia) in rats [126]. Years later, the use of ketamine/xylazine was reported to reduce the number of SDs as well as the infarct volume in a photothrombotic model of stroke [137]. The beneficial effects of ketamine as a potential candidate to reduce the appearance of these slow-propagating waves were also assessed after MCA occlusion in different animal models [127, 133]. Specifically, in mice, the generation of SD clusters was induced by microinjection of KCl after distal MCA occlusion. This study suggested that ketamine administration decreased intracellular Ca^2+^ accumulation following a SD wave, a mechanism that could reduce neuronal swelling and, consequently, edema formation after an ischemic insult [127]. Also in mice, ketamine prevented the infarct growth closely linked to SDs in the delayed phase after ischemia [186]. Similarly, the administration of ketamine at human therapeutic doses in swine subjected to MCA occlusion led to a reduction in SD incidence and propagation [133].

### Clinical evidence supporting CSD-targeting therapies

In 2009, a small cohort of TBI and ICH patients was treated with ketamine to inhibit SDs, in addition to successfully restoring ECoG activity [132]. A substantially larger group of patients (*n* = 115), including MHS patients, was later analyzed in a retrospective study evaluating the influence of several drugs commonly administered in intensive care units (i.e., midazolam, propofol, fentanyl, sufentanil, ketamine, and morphine) on SD onset. As expected, ketamine infusion decreased the number of isoelectric SDs, in contrast to midazolam, whose administration led to a higher number of SD clusters [72]. Furthermore, Carlson et al. [26] also confirmed that the use of ketamine in a wide range of doses commonly chosen for sedation inhibits SD appearance in TBI and subarachnoid hemorrhage (SAH) patients [26]. Recently, in a MHS patient experiencing non-treatable SDs, linked to exacerbation of brain damage and unconsciousness, clinicians made the decision to initiate ketamine treatment based on prior evidence. No further SDs were observed once ketamine was adjusted to sedative doses, and ECoG activity was restored in peri-infarcted tissue [28].

However, there are other therapeutic options that have been considered, despite most of them being still at pre-clinical stages. One of these alternative avenues is the antiepileptic treatment topiramate, which provides beneficial effects also in preventing migraines. More in detail, chronic treatment with topiramate daily for 7 weeks decreased the susceptibility to SDs in WT and familial hemiplegic migraine type 1 mutant mice, after MCAO followed by KCl or electrical SD stimulation [47]. Recently, Wang et al. [170] described that the same drug reduced PIDs, leading to recovery of somatosensory evoked potentials in the forelimb area of the cortex, as well as a reduction of infarct volume and neuroinflammatory markers 24 h after photothrombotic ischemia [170]. Using the same stroke model, the benefits of systemically antagonizing adrenergic receptors were evaluated. Specifically, propranolol, prazosin, and atipamezole showed promising results in accelerating extracellular K^+^ clearance, leading to a reduced infarct area and better motor functional outcomes [110].

In addition to adrenergic receptors, other receptors were considered interesting targets to inhibit SDs. Despite the prostaglandin F receptor having traditionally been associated with the exacerbation of neurodegeneration, its role in regulating CBF hemodynamics as well as SD occurrence had not previously been investigated after brain injury. Thus, Varga et al. [165] observed that following bilateral occlusion of the common carotid artery, pharmacological antagonism of the prostaglandin F receptor ameliorated the perfusion of ischemic regions and reduced SDs in the rat cortex, leading to decreased programmed cell death [165].

Another interesting target is the Sigma-1 receptor. It regulates Ca^2+^ trafficking between the endoplasmic reticulum and mitochondria and modulates the expression of certain K^+^ channels [152]. Besides, N,N-dimethyltryptamine (DMT) is naturally present in some plants, and its controversial psychedelic effects have not masked its potential beneficial effects, reducing SD after hypoxia [151] and ischemia [115], protecting cells and reducing infarct volumes, respectively. After bilateral occlusion of the common carotid artery, DMT administration reduced SD amplitude, as well as the cumulative duration of them in comparison to controls [152].

Moreover, either inositol triphosphate or transient potential receptors play relevant roles in the regulation of Ca^2+^ dynamics. Recently, Fernández-Serra et al. [51] described that aminoethoxydiphenyl borate (2-APB), a non-selective antagonist of both receptors, reduced the ischemic region size as well as improved somatosensory evoked potentials after a permanent model of ischemic stroke. This effect was linked to a decrease in PID duration accompanied by better cerebral perfusion in the metabolically active penumbra [51].

In addition, to reduce intracellular Ca^2+^ accumulation, nimodipine, a dihydropyridine L-type Ca^2+^ channel blocker, has emerged as an interesting candidate to prevent CSD onset in MHS patients. Notably, nimodipine is already approved for oral or intravenous administration in SAH patients. It is described that nimodipine has the capability to switch the neurovascular coupling response to the physiological one [41]. Studies in rodents have shown that nimodipine administration reduces both CSD appearance and the extent of secondary injury [44]. These findings were further corroborated in a small cohort of SAH patients, where nimodipine treatment was associated with reduced depression of ECoG activity [166]. With the aim of inhibiting persistent SDs that exacerbate ischemic damage and preserving function of metabolically active penumbral tissue, Toth et al. (2020) developed some pH-sensitive hydrophobic nanoparticles. Nimodipine is encapsulated within these nanoparticles, and in response to the acidification of the ischemic environment, it is selectively released in affected areas. When applied directly to the brain cortex after global forebrain ischemia, nimodipine-loaded nanoparticles enhanced neuronal repolarization and hyperemia and significantly reduced both the duration of SDs and the extent of tissue acidosis [163].

Recently, one of the enantiomers of the well-described antivertigo drug S-meclizine has been associated with an increase in glucose utilization in the penumbra but not in the ischemic core when administered before transient MCAO. The switch from oxidative phosphorylation to glycolysis delays the anoxic depolarization onset and reduces brain infarct. It has been considered an interesting approach as a prophylactic for patients at risk of ischemic stroke or myocardial infarction [98].

Although blocking receptors and modulating downstream signaling pathways remain the most common strategies to counteract SDs, it is important to consider that neurons also exhibit imbalances in both homeostasis and activity. These alterations in brain cells contribute to excitotoxicity, PIDs propagation, and ultimately to secondary brain injury. Based on this, Wang et al. [171] selectively inhibited the activity of a subset of excitatory forebrain neurons during the early stages after ischemic insult in order to improve stroke outcomes. To this end, mice were genetically modified to express hM4Di, a Designer Receptors Exclusively Activated by Designer Drugs (DREADD), in forebrain excitatory neurons. Upon chemogenetic activation of these receptors by clozapine-N-oxide after transient MCAO, evoked synaptic responses in the motor cortex were attenuated, resulting in improved neurological function and reduced infarct volume. In addition, KCl-induced CSDs were prevented after administration, suggesting that targeted inhibition of a subset of excitatory neurons may prevent the propagation of secondary brain damage [171].

In line with the strategy of modulating neuronal excitability, vagus nerve stimulation (VNS) is an FDA-approved practice for epilepsies, depression, and migraines. The vagus nerve consists of an extensive network of fibers that influence neuroendocrine and immune functions through interactions with various brain regions. Notably, the reduction of migraine headaches has been strongly associated with decreasing the susceptibility to SDs [29]. Furthermore, it was demonstrated that VNS plays a role in reducing infarct volume and improving neurofunctional outcomes after stroke in some animal models [75, 183]. More recently, both invasive and non-invasive VNS approaches have been reported to decrease the frequency of SD appearance after ischemia. While these interventions effectively reduced infarct volume, mostly in the cortical area, their effects on sensorimotor outcomes were less pronounced than expected [101]. Encouraged by these results, a prospective clinical trial (NOVIS) was launched in 2020 at Leiden University Medical Centre. The study is recruiting ischemic stroke patients to evaluate the therapeutic potential of non-invasive VNS. However, the recruitment of patients was delayed due to COVID-19, and the results of this study are not yet available. Another pivotal study (VNS-REHAB) investigated the effect of VNS combined with rehabilitation on upper limb motor recovery in ischemic stroke patients, showing favorable outcomes [32].

In summary, despite there being a wide range of therapeutic approaches that have been tested at preclinical levels, further translational efforts are needed, as ketamine remains the most used and clinically accepted treatment by clinicians to counteract SDs. However, experts from different clinical areas that comprise the COSBID reported that a complementary approach to pharmacological treatment may involve early modulation of systemic physiological variables closely associated with these pathological waves (e.g., hypotension, decreased perfusion pressure, hypoglycemia, and high corporal and brain temperature, among others) [71]. Subsequent studies confirmed the relevance of these factors. They showed that alterations in systemic parameters after ischemic injury significantly influence SD dynamics [28, 138]. Supporting this hypothesis, Kentar et al. [81] showed that hypothermia, when applied after MCAO in swine, effectively reduced both the incidence and propagation of SDs, as well as the volume of infarcted tissue, compared to normothermic conditions [81].

### CSDs as predictors of reperfusion failure

Although numerous pharmacological strategies have been tested over the past decades to prevent ischemic damage and neurological dysfunction, recanalization of the occluded vessel remains the first-line strategy when a patient presents to the emergency department with stroke symptoms [146]. However, sometimes patients submitted to a successful recanalization, corroborated by angiography, still present poor outcomes and die in the worst of the cases [161]. This phenomenon is diagnosed as no-reflow, or futile recanalization, and it is closely associated with aged people and poor collateral circulation. Generally, the pathological mechanisms that trigger this situation typically include distal clot fragmentation (i.e., embolization), pericyte constriction, or neutrophil obstruction of capillaries following large vessel occlusion [16, 162]. Importantly, the appearance and propagation of CSDs seem to play a role in the success of recanalization, being considered a predictor of a no-reflow event [162]. In their study, Törteli et al. [162] monitored the SD profile as well as the CBF after bilateral clamping of CCAs and reported that reperfusion failure was produced in hemispheres in which SDs appeared upon the release of the clamps. This was closely related to neuronal necrosis that resulted in worse somatosensory neurological deficits. The authors hypothesized that the propagation of SDs was prominent in those animals that had an incomplete circle of Willis [162]. This anatomical variation is present in more than 50% of adults, an alteration that can be particularly problematic after the occlusion of a blood vessel, as the recruitment of collaterals compensates for the reduced perfusion [161]. The influence of collateral circulation, especially through leptomeningeal pial vessels, to prevent futile recanalization after thrombin microinjection was also detailed in [16]. Basically, inadequate collateral circulation was linked to rapid and excessive reperfusion, resulting in greater tissue damage and more severe clinical deficits [16]. As previously described by Binder et al. [17], SDs are preceded by a brief hyperemic response, followed by a strong hypoperfusion (i.e., spreading oligemia), primarily mediated by capillary constriction. Then, it is suggested that SD-mediated reperfusion failure should be linked to this vasoconstriction, which is presumably caused by 20-HETE metabolized from arachidonic acid [161, 162]. Thus, understanding the mechanisms behind futile recanalization and recognizing spreading depolarizations as a potential contributor could open the way to novel therapies to improve stroke outcomes [162].

## Monitoring techniques

As discussed in previous sections, the interruption of blood supply caused by stroke leads to complex alterations in the brain, requiring a comprehensive and integrative approach to monitoring these changes. The complexity of ischemic brain injury, coupled with its dynamic progression, underscores the importance of adopting a multimodal monitoring approach by integrating information from various techniques to better understand and manage these pathophysiological events (Table 1). In clinical practice, the most widely employed techniques for monitoring brain function following injury include intracranial pressure (ICP), CPP, O_2_ availability (measured as local tissue partial pressure of O_2_ [ptiO2]), and scalp electroencephalography (EEG). However, while these techniques provide critical insights into key physiological parameters, they often fall short in detecting the subtle, yet significant alterations associated with CSDs.

Monitoring SDs has major diagnostic relevance because these events are closely associated with lesion progression in ischemia and traumatic brain injury [4]. SDs serve as a dynamic biomarker of ischemic lesion evolution. Their detection allows identification of metabolically compromised areas and assessment of ongoing tissue damage. Importantly, SD shape, frequency, and duration also provide prognostic information. For example, longer SD-induced depressions correlate with infarct growth in malignant hemispheric stroke [88]. Real-time SD monitoring can therefore serve not only for prognostication but also for evaluating the brain’s response to neuroprotective interventions, potentially guiding timely therapeutic adjustments.

This section reviews the state-of-the-art methods for monitoring SDs, encompassing electrical, optical, ultrasound, and magnetic-based technologies.

### Electrophysiological approaches

Electrographic techniques have recently advanced to incorporate intracranial ECoG as a reliable method for CSD monitoring. Unlike scalp EEG, intracranial ECoG addresses the challenges of limited spatial resolution and sensitivity, enabling more accurate detection of these events [37, 76]. By bypassing the skull, ECoG eliminates signal distortion caused by skull filtering, thereby offering a more precise and effective approach despite its invasive nature.

The current gold standard for monitoring CSDs is subdural ECoG utilizing a linear electrode strip comprising six platinum/iridium contacts, each with an exposed surface area of 4.2 mm^2^ and spaced 10 mm apart [37]. The electrode strip is placed subdurally on the cortical surface to monitor brain tissue that is at risk of secondary injury. It is commonly used in conditions such as aneurysmal subarachnoid hemorrhage, traumatic brain injury, intracerebral hemorrhage, and malignant hemispheric stroke, with placement tailored to the affected brain region [37]. However, the performance of ECoG electrodes is frequency dependent, and their ability to fully capture the electrical phenomena associated with CSDs, such as DC shifts and infralow oscillations (< 0.1 Hz), is compromised. This problem arises because platinum electrodes are polarizable. Polarization causes potential drift, especially at low frequencies, which overlaps with brain signals [99]. As a result, clinical ECoG monitoring has relied on AC-coupled amplifiers, which exclude infra-slow signals. More recently, advancement in electronics instrumentation has enabled the implementation of DC-coupled amplifiers, which overcome the amplifier saturation issues produced by electrode polarization voltages. Several studies propose its use for continuous ECoG monitoring [40, 66, 116], offering the potential to record low-frequency and DC potentials, while considering the limitations of electrodes for performing high-fidelity DC-coupled recordings such as artefacts which result in unwanted DC shifts and infra-slow signal filtering [65]. Although such studies remain constrained to a small number of electrodes with millimeter-sized surfaces, these advancements are promising for capturing the full spectrum of electrophysiological changes associated with CSDs and related phenomena.

Traditionally, pre-clinical studies have used liquid-filled glass micropipettes with Ag/AgCl wires as the gold standard for DC-coupled recordings. However, these are limited to one or a few recording sites, restricting spatial mapping. Recent innovations in implantable multielectrode arrays, made possible by advances in microfabrication and materials science, now allow recordings with higher spatial resolution and more recording sites, greatly improving data quality [117, 145, 156]. Despite these advancements, similar to ECoG, most microelectrode array technologies remain susceptible to DC offsets and low-frequency drifts due to electrode polarization [99]. This drift is directly related to electrode impedance [99], which increases as electrode size decreases. As a result, there is a trade-off between achieving high spatial resolution and maintaining reliable DC signal recordings. Future research could direct towards optimizing electrode materials, geometries, and surface treatments to minimize potential drift and enable accurate, stable recordings of DC signals while maintaining high spatial resolution. A recent study introduced high-density cortical µECoG arrays to monitor SDs and long-term stroke characteristics in awake, non-anesthetized animals [121]. In this study, an electrode surface treatment based on electroplating platinum iridium (PtIr) on the gold contacts (200 µm in diameter) was applied to further reduce the impedance (~ 600–700 kΩ at 0.05 Hz). While this modification allowed recordings in the infraslow frequency range, the data presented in the study revealed significant attenuation and distortion in the recorded signals compared to the gold standard micropipette recordings of CSDs.

As an alternative to passive electrodes, in the last years, active signal transducers based on graphene solution-gated field effect transistors (gSGFETS) have been studied for full-bandwidth electrophysiological recordings [18, 106]. Graphene is proposed as an efficient bio-transducer material due to its high electrochemical stability, biocompatibility, and high charge sensitivity [54, 69, 106]. A gSGFET features a graphene sensing area that serves as a channel, connecting two metal terminals (drain and source). The graphene channel interfaces directly with an electrolyte solution or biological tissue, which contains a reference electrode functioning as the gate terminal. The unique properties of gSGFETs enable the modulation of the graphene channel conductivity by brain electrical activity, thereby controlling the current between the drain and source terminals. This transistor-based configuration offers several distinct advantages for electrophysiological recordings, including local preamplification of signals, which potentially reduces noise, stable gain across a broad frequency range, and the potential for multiplexing technology [54, 69]. These features make gSGFETs highly suitable for advanced neurophysiological studies. Notably, the combination of the direct DC coupling capability and high electrochemical stability of graphene led to the recent demonstration of high-resolution mapping of infralow (< 0.1 Hz) brain signals with gSGFETs [18, 106]. The reported recordings achieved a fidelity comparable to liquid-filled glass micropipettes. Importantly, they also overcame spatial resolution limitations, enabling better investigation of infralow activity such as CSDs. Moreover, gSGFETs can be embedded in highly conformable flexible substrates, allowing mapping of full-bandwidth brain signals using multiplexing technology [31, 55, 135]. This multiplexing capability paves the way for scaling up gSGFET technology towards large-scale, high-density recording arrays that could resolve neural activity at high resolution over large brain regions while acquiring signals across a wide frequency spectrum [179].

### Imaging approaches

Besides electrophysiological recordings, other techniques have been used to monitor CSDs (Table 2). CSDs can be detected by their distinct changes in electrical activity as well as changes in CBF, which reflect the intricate relationship between neuronal activity and vascular responses, as mentioned earlier, known as neurovascular coupling [36, 141]. Consequently, optical imaging techniques that measure blood flow have emerged as effective alternatives to electrographic methods for detecting CSDs. Among these, laser speckle imaging (LSI) has been demonstrated as a reliable approach for capturing hemodynamic responses with high spatiotemporal resolution in both intraoperative and preclinical studies [175]. LSI employs the random interference pattern of scattered laser light, called a speckle pattern, to measure blood flow. When blood flow increases, the moving particles cause faster intensity fluctuations in the speckle pattern, leading to blurring during camera exposure. By quantifying this blurring, spatial maps of relative blood flow can be created, providing high spatiotemporal resolution. First described for CSD monitoring in 2001 [46], LSI has since proven effective for reliably tracking the spatial propagation of CSDs and has significantly enhanced our understanding of their dynamics [92, 114, 141, 147, 154].

**Table 2:** Comparison of state-of-the art techniques for monitoring cortical spreading depressions (CSDs) associated to brain pathophysiology

Technique	Measured parameter	Spatial resolution	Advantages	Limitations	Ref
Liquid-filled pipettes	Extracellular electrical activity	< Neuron—column	Reliable DC signal recording	Restricted to few measurement points Not clinically transferable Not suitable for chronic recordings	[79]
Scalp EEG	Extracellular electrical activity	Area—whole brain	Non-invasive Wide clinical availability	Limited spatial resolution Signal attenuation due to skull filtering Poor sensitivity	[27, 45, 76]
Subdural ECOG	Extracellular electrical activity	Hypercolumn—area	High-quality signal Good spatiotemporal resolution	Invasive Poor sensitivity for infraslow activity detection (artifacts, drifts)	[37, 40, 67, 76, 116]
Micro-electrode arrays	Extracellular electrical activity	Column—area	High-quality signal High spatiotemporal resolution	Invasive Poor sensitivity for infraslow activity detection (artifacts, drifts, intrinsic filtering)	[117, 121, 145, 156]
GSGFET	Extracellular electrical activity	Column—area	Reliable DC signal recording Stable gain across a wide range of frequencies High spatiotemporal resolution	Invasive	[18, 55, 69, 106, 135]
Ca2^+^ imaging	Intracellular Ca2+ concentration	Neuron—area	High spatial resolution Cell-specific monitoring Wide-field imaging	Not clinically transferable Not suitable for chronic recordings	[14, 103]
LSI	rCBF	2D area	High spatiotemporal resolution Wide-field imaging	Limited depth penetration Indirect measurement Limited to experimental models Not suitable for chronic recordings	[46, 92, 114, 147, 154]
ISOI	rCBF		High spatial resolution Wide-field imaging	Limited depth penetration Indirect measurement Limited to experimental models Not suitable for chronic recordings	[13, 172, 185, 189]
fUS	rCBF		High spatiotemporal resolution Deep tissue penetration Wide-field imaging	Indirect measurement	[22, 78, 188]
MEG	Magnetic fields induced by neuronal activity	Area—whole brain	Non-invasive Whole-brain coverage	Limited spatial resolution Poor sensitivity Not suitable for chronic recordings	[20, 174]

Another optical technique that has been reported for CSD monitoring is intrinsic signal optical imaging (ISOI). ISOI is a functional imaging technique which measures neural activity by detecting changes in hemodynamic responses in brain tissue, including blood flow and O_2_ consumption. In particular, it measures changes in the light reflectance related to local changes in the oxy- to deoxy-hemoglobin concentrations and local CBF changes [111]. It has been extensively used to study the spatiotemporal propagation of CSDs [13, 172, 175, 185, 189]. However, this technique faces several challenges, including limited penetration depth, reduced imaging precision, and susceptibility to biological noise (e.g., vasomotion signals) and motion artifacts such as heartbeat and respiration-induced movement [189]. In addition, not every CSD is associated with a detectable ISOI signal change, highlighting a limitation in its sensitivity [175].

Within the optical domain, optogenetic-based fluorescence imaging has proven to be a powerful technology able to monitor and manipulate brain activity with high spatiotemporal resolution in a more direct way than LSI and ISOI. Typically, in optical functional imaging, a genetically encoded indicator sensitive to neural activity is introduced to the target cells. When an action potential occurs in a neuronal cell, the voltage-gated Ca^2+^ channels open, leading to an influx of Ca^2+^ ions and a subsequent increase in the intracellular Ca^2+^ concentration. Genetically encoded Ca^2+^ indicators respond to the increase in Ca^2+^ concentration by emitting fluorescence when excited at a specific wavelength [100]. The use of Ca^2+^ indicators has shown promising results for monitoring CSD wave propagation in stroke animal models [14, 103]. However, these techniques are not suitable for clinical use as they rely on genetic modifications.

Besides optical imaging, other domains such as ultrasound and magnetic modalities offer complementary approaches for CSD monitoring, each with distinct advantages and limitations. Among these, functional ultrasound imaging (fUS) stands out for its ability to image the hemodynamic response across the whole-brain microvasculature with high spatiotemporal resolution [105]. Notably, it provides advantages over optical techniques, such as superior tissue penetration, and has demonstrated potential in preclinical studies for visualizing the propagation of CSDs and their associated vascular responses [22, 78, 188]. On the other hand, magnetoencephalography (MEG) emerges as a non-invasive method that records the magnetic fields produced by neuronal activity with high temporal resolution. However, while its use for CSD detection has been reported in a few studies [20, 174], its suitability for monitoring slow potential changes remains limited due to low sensitivity. In addition, MEG suffers from poor spatial resolution, making it challenging to accurately localize the propagation of CSDs.

### Multimodal monitoring strategies

The use of multimodal methods combining different modalities such as electrographic and hemodynamic capabilities holds great promise for helping decipher the intricate changes associated with spreading depolarizations [105]. Recent studies have shown concurrent compatibility of thin-film graphene devices allowing DC-coupled mapping and optical imaging [106] or functional ultrasound imaging [188]. The approach in this last work is particularly promising as it allows high spatiotemporal mapping of both electrographic and vascular changes associated with CSD, enabling a better understanding of its dynamics in both surface and deep brain structures.

## Conclusions and future perspectives

Cumulative evidence establishes CSDs as critical events in the progression of brain injury, with outcomes highly dependent on the metabolic and vascular status of affected tissue. As a result, neuroprotective interventions targeting CSDs, such as NMDA receptor antagonists, anesthetics, and anti-migraine drugs, have shown promise in mitigating the damage induced by CSDs and improving patient outcomes. These findings underscore the importance of not only understanding the mechanisms driving CSDs but also developing strategies to counteract their harmful effects. This knowledge opens new avenues for targeted therapies, precision monitoring, and diagnostic innovations aimed at improving the management of stroke, traumatic brain injury, and other neurological conditions associated with CSDs. However, despite advancements in electrical, optical, ultrasound, and magnetic-based technologies for CSD monitoring, these methods still require refinement to ensure reliable detection, assessment, and to allow their use in the clinics. Enhancing these technologies will facilitate the precise tailoring of interventions to the specific pathophysiological context, enabling clinicians to mitigate the devastating consequences of CSDs and significantly improve care for patients with acute brain injuries.

## Data Availability

Not applicable.
